# Periosteal chondroma of the femur: A case report and review of the literature

**DOI:** 10.3892/ol.2015.2889

**Published:** 2015-01-23

**Authors:** KAI ZHENG, XIUCHUN YU, SONGFENG XU, MING XU

**Affiliations:** Orthopedic Department, General Hospital of Jinan Military Commanding Region, Jinan, Shandong 250031, P.R. China

**Keywords:** periosteal chondroma, femur, differential diagnosis

## Abstract

Periosteal chondroma is a rare benign cartilage tumor located on the cortical bone, which may be mistaken clinically and histologically for other and more common tumors in this location. The current study reports the case of periosteal chondroma located in the distal femur of a 14-year-old female. A non-tender swelling, 5×4 cm in diameter, was identified on computed tomography, a radiological study of which revealed an overhanging edge and a radiolucent shadow with stippled calcification in radiographs and a lobular heterogeneous mass in magnetic resonance imaging. Cytological examination of the excision biopsy revealed cellular pleomorphism and binucleate cells. The patient underwent en bloc resection of the tumor and covering periosteum, and the histological diagnosis was subsequently determined to be periosteal chondroma. The present study also reviews nine previously reported cases of periosteal chondroma in the femur, with a discussion of the demographic characteristics, imaging features, differential diagnosis and treatment of bone tumors in this location. This study aims to inform clincians that periosteal chondromas may occur in the distal femur although osteochondromas are more common and to discuss making a differential diagnosis between periosteal chondroma and other bone tumors.

## Introduction

Periosteal chondroma is a rare benign cartilaginous tumor that occurs under or in the periosteum on the surface of cortical bone. It was first described by Lichtenstein and Hall (1) in a report of six cases. Periosteal chondromas account for <2% of all chondromas and may occur in adults or children (2), however, the disease is predominantly identified in patients <30 years of age, with the highest frequency between the ages of 10 and 20 years (3). It is typically observed in the metaphyses of long bones beneath the periosteal membrane, particularly the proximal humerus and tubular bones of the hands (2,4). A number of studies have also reported erosion of the spine, clavicle and costal cartilage (2,7,8). The diagnosis of periosteal chondromas is primarily based on histological characteristics. Although the genetic mechanisms of the disease remain unclear, genetic rearrangement in chromosome 12 has been reported to be associated with this disease (5). Treatment for periosteal chondroma includes intralesional, marginal and en bloc resection and the recurrence rate is 3.6% (2).

The current study reports a case of periosteal chondroma located in the distal femur, and reviews the literature with regard to clinical, radiological, histopathological and treatment criteria, in addition to discussing the differential diagnosis of this condition. Written informed consent was obtained from the patient’s family.

## Case report

**Figure 1:**
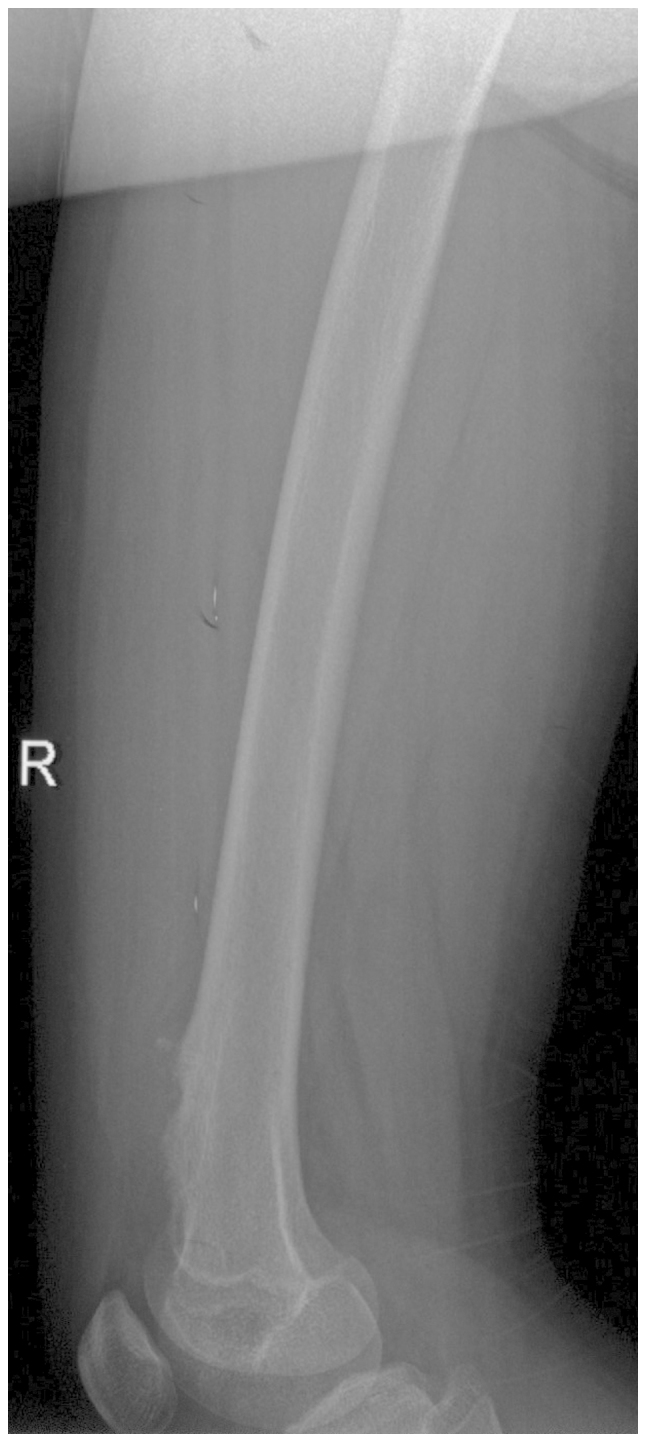
Radiography showed a large radiolucent shadow with stippled calcification.

**Figure 2:**
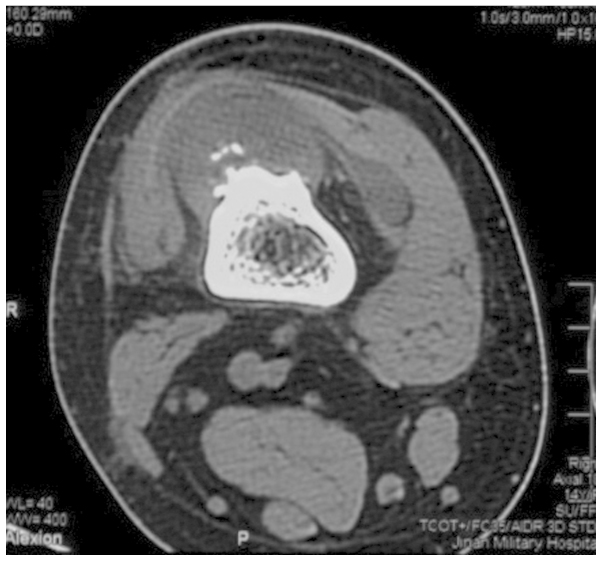
Computed tomography revealed a solid tumor of 5×4 cm in diameter and associated with the right distal femur bone, with abundant specks of calcification.

**Figure 3:**
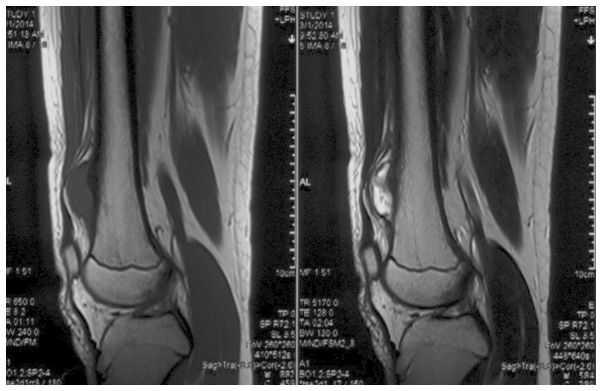
Magnetic resonance imaging revealed a lobular heterogeneous mass arising from the femur periosteum.

A 14-year-old female presented to the Orthopedic Department of the General Hospital of Jinan Military Commanding Region (Jinan, China) with a two*-*month history of a gradually increasing mass in the right lower thigh. No history of trauma or episodes of pain similar to that observed in other bone tumors, such as continuous pain or pain at night, were noted. Firm, non-tender swelling in the distal thigh was observed upon physical examination, with normal overlying skin and no neural deficit. Radiographs revealed an overhanging edge and a radiolucent shadow with stippled calcification (Fig. 1). Computed tomography (CT) imaging showed a solid tumor of 5×4 cm in diameter, with specks of calcification, which was associated with the right distal femur bone (Fig. 2). Magnetic resonance imaging (MRI) revealed a lobular heterogeneous mass arising from the femur, which was hypointense on T1-weighted images and hyperintense on T2-weighted images (Fig. 3). No aggressive radiological appearance was observed, and chromosomal analysis was unremarkable. Cytological examination of the excision biopsy revealed cellular pleomorphism and binucleate cells.

**Figure 4:**
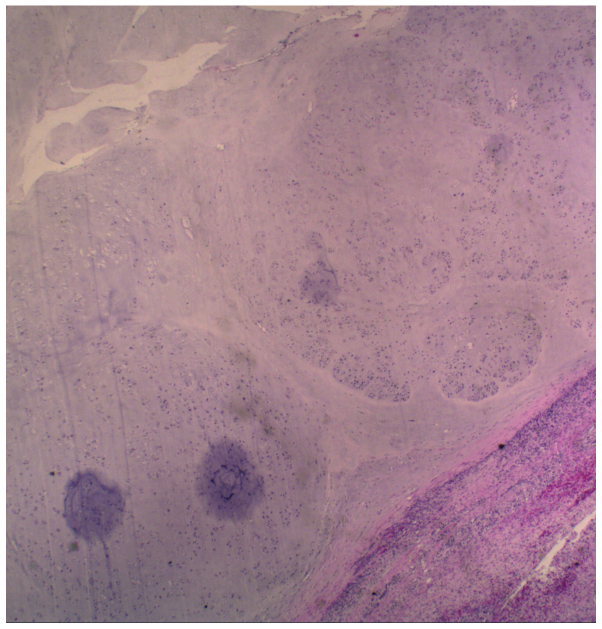
Lobules of hyaline cartilage composed of chondrocytes with endochondral ossification (hematoxylin and eosin staining; magnification, ×200).

Although cytological findings indicated a benign tumor, a diagnosis of chondrosarcoma could not be excluded. Therefore, an extended resection was performed; the tumor was excised en bloc, including margins of normal bone, and ipsilateral limb fibula transplantation was used to repair the cortical defect. Microscopic examination revealed lobules of hyaline cartilage composed of chondrocytes, with foci of endochondral ossification and calcification (Fig. 4). The lesion showed hypercellularity, however, cytological atypia was not observed. Additionally, no penetration to the medullary cavity and surrounding soft tissues was identified. A diagnosis of periosteal chondroma was therefore determined.

The patient’s surgery was successful and six weeks later, the patient walked with the aid of a brace. At follow-up, radiographs revealed that healing had occurred following the bone graft and no new tumor tissue was observed. No further treatment was required.

## Discussion

Periosteal chondroma is an uncommon benign tumor of the hyaline cartilage (6). It is slow-growing and frequently erodes the cortex. The lesion is generally observed between the ages of 10 and 30 years with equal gender distribution (2–4). The patient in the present study was 14-year-old female with a lesion in the right distal femur; previous cases of periosteal chondroma occurring in the femur are summarized in Table I (2,3,9,10).

Of the 10 reviewed cases of periosteal chondroma of the femur, five were males, four were females and one had no reported gender data. The mean age at the beginning of treatment was 14.67 years (range, 6–26 years), excluding one case in which no accurate age was reported. With regard to the location of the tumor, six cases involved the distal femur, three involved the proximal femur, and one involved the middle femur.

Evaluation of the clinical course of periosteal chondroma may aid in its diagnosis. Typically, the first symptom is localised swelling, followed by moderate and prolonged pain (~6 months) (3). Of the reviewed cases, five patients exhibited this pattern of symptoms for a long duration (≥10 months) (mean, 18.1 months; range, 1–108 months). The presence of pain is a significant clinical feature in the evaluation of cartilaginous tumors; in adults with a histologically low-grade cartilaginous tumor, we hypothesize that pain may indicate aggressive biological behavior. However, in the reviewed cases, the presence of pain was not associated with treatment procedure or clinical effect.

Radiographic features of periosteal chondroma may vary between cases. The mass typically arises from the surface of bone, exhibiting scalloping of the cortex and a well-defined margin between the tumor and bone (3). Tumor sclerosis in this type of tumor is common and may include overhanging edges and soft-tissue masses with a variable pattern of calcification and ossification. Of the nine cases for which radiographic pattern data was provided, one showed clear scalloping of the cortex, two exhibited overhanging edges, three showed sclerosis, two showed calcification or ossification of the cartilaginous matrix and one patient exhibited no calcification or ossification of the cartilaginous matrix. In the current case, CT imaging revealed a soft tissue mass of iso and high density, containing stippled calcifications and was associated with the destruction of local regions of bone. MRI showed a sharply delineated subperiosteal lobulated mass at the bone surface, consisting of a matrix with a bright signal, a hypointense lining on T2-weighted images and isointense signal relative to muscle on T1-weighted images. The absence of CT and MRI data in the reviewed cases prevents further speculation with regard to the use of these imaging modalities for the diagnosis of this condition.

Histopathologically, periosteal chondromas exhibit a prominent lobular arrangement of hyaline cartilage extending from the periosteum into the adjacent cortical bone (4). They are typically hypocellular, however, increased cellularity with nuclear pleomorphism, binucleation, and multinucleation may be observed (2–4). The presence of hypercellularity and nuclear atypia, which often indicates malignancy, may lead to difficulties in the differential diagnosis of such cases.

The differential diagnosis of periosteal chondroma in this location includes chondrosarcoma, osteochondroma and periosteal osteosarcoma. Compared with periosteal chondromas, chondrosarcomas are generally greater in size and occur in older patients (>50 years of age); extension into the soft tissue may also be observed (3). Osteochondromas are more commonly detected in the femurs of adolescents, in contrast with periosteal chondromas, which typically occur in young adults (11). Osteochondromas may also be distinguished by the presence of a dense osteoid formation in the cortex and medulla of the mass, and by the continuation with its originating bone (12). Periosteal chondrosarcoma shows popcorn calcifications on radiographs, which present as a collection of scalloped radiolucencies, each with a sclerotic margin, whereas periosteal osteosarcoma commonly exhibits perpendicular spicules of calcification (13). Periosteal osteosarcomas are slow-growing, and primarily arise beneath the periosteum in the distal femur, inducing new bone formation. This is observed as a radiolucent lesion on the bone surface with perpendicular striae and a peripheral Codman’s triangle (14). Lace-like, malignant osteoids may be detected, however, chondroid areas with high-grade anaplastic cells dominate the histological appearance (14). Differentiating between these conditions may necessitate an excision biopsy; however, it may also be difficult to distinguish low-grade chondrosarcoma from chondroma on the basis of histology (13).

A previous literature review revealed that, among 165 cases of periosteal chondroma, six reported local recurrence (a recurrence rate of 3.6%) (2). Of the cases of periosteal chondroma in the femur, no recurrence was noted during postoperative follow-ups (range, 6 months to 22 years; mean, 7.22 years). The treatments for periosteal chondroma of the femur included marginal excision, en bloc resection and curettage. In the cases reviewed in the current study, two patients were treated by en bloc resection, four by marginal excision, three by curettage and one by hip disarticulation following incisional biopsy and radiotherapy. Marginal excision and curettage are preferable options if the diagnosis is certain prior to surgery; otherwise, en bloc resection may be a more beneficial treatment strategy. Therefore, the results of this study may increase current understanding with regard to the diagnosis of periosteal chondroma in the femur and the selection of suitable treatments.

## Figures and Tables

**Table I:** Clinical characteristics of 10 patients with periosteal chondroma in the femur.

Author (reference)	Gender/age, years	Site	Symptoms	Duration of symptoms, months	Radiographic pattern	Treatment	Follow-up, years
Boriani *et al* (3)	M/11	PF	Pain, mass	24	Overhanging edges	Marginal excision	22
	M/26	DF	Pain	10	Calcification of matrix	Marginal excision	4
	M/6	DF	Pain	12	Scalloping	Marginal excision	3
	M/18	MF	Pain, mass	10	Calcification of matrix	Incisional biopsy, radiotherapy, hip disarticulation	22
Lewis *et al*(2)	F/9	PF	Pain	5	Sclerosis	Curettage	3
	M/19	DF	Pain, mass	4	No calcification of matrix	Curettage	3
	NP	DF	Pain	5	NP	Marginal excision	3
Tillich *et al* (9)	F/8	PF	Pain	1	Sclerosis	Curettage	NP
Wheelhouse and Griffin 10)	F/21	DF	Mass	108	Sclerosis	En bloc resection	4.5
Zheng (current)	F/14	DF	Swelling	2	Overhanging edges	En bloc resection	0.5

M, male; F, female; PF, proximal femur; DF, distal femur; MF, middle femur; NP, not provided.
